# Actin filaments drive spindle positioning in Arabidopsis meiosis II

**DOI:** 10.1111/nph.70625

**Published:** 2025-09-29

**Authors:** Yingrui Ma, Hua Jiang

**Affiliations:** ^1^ Leibniz Institute of Plant Genetics and Crop Plant Research Gatersleben 06466 Germany; ^2^ Institute for Biochemistry and Biology, University of Potsdam Karl‐Liebknecht‐Str. 24‐25 14476 Potsdam‐Golm Germany

**Keywords:** actin polymerization, ploidy, spindle separation, the organelle band, unreduced gamete

## Disclaimer

The New Phytologist Foundation remains neutral with regard to jurisdictional claims in maps and in any institutional affiliations.

## Introduction

Meiosis is a unique cell division process for sexual reproduction in eukaryotes. A typical meiotic cell cycle contains one round of DNA duplication followed by two rounds of chromosome division, meiosis I and meiosis II (Klug *et al*., 2013; Mercier *et al*., 2015). Meiosis I divides homologous chromosomes, whereas meiosis II divides sister chromatids. In monocots, successive cytokinesis forms a cell wall after meiosis I, physically separating spindles in meiosis II (Brownfield & Kohler, 2011; De Storme & Geelen, 2013a,b). By contrast, dicots undergo simultaneous cytokinesis, where an organelle band composed of various organelles serves as a physical barrier (Brownfield *et al*., 2015). The organelle band ensures proper spindle positioning and chromosome segregation at the second meiotic division (Brownfield *et al*., 2015). The endomembrane protein JASON (JAS) is critical for the stability of the organelle band in dicots, like Arabidopsis and potatoes (Erilova *et al*., 2009; De Storme & Geelen, 2011; Brownfield *et al*., 2015; Clot *et al*., 2024). Disruption of the organelle band leads to misoriented spindles and further produces dyads and triads after cytokinesis (De Storme & Geelen, 2011; Brownfield *et al*., 2015).

Spindle positioning is decided differently in plants and animals. In most eukaryotic cells, spindle microtubules connect chromosome kinetochores to microtubule organization centres (MTOCs) at both poles (Sanchez & Feldman, 2017). In mammalian cells, centrosomes, as the primary MTOCs, assemble microtubules, actin, and motor proteins to the nuclear envelope, facilitating pole determination (Wu & Akhmanova, 2017). By contrast, higher plants lack centrosomes, suggesting a different spindle orientation mechanism (Yi & Goshima, 2018). The spindle orientation is especially critical in the simultaneous meiotic cell cycle since the cell wall is not formed until the end of telophase II. Interestingly, mammalian oocytes also lack centrosomes, where spindle positioning relies on a meshwork of actin filaments that actively guides the spindle positioning and mobility (Azoury *et al*., 2008; Li *et al*., 2008; Schuh & Ellenberg, 2008; Field & Lenart, 2011; Mori *et al*., 2011; Mogessie *et al*., 2018). Given the similar absence of centrosomes in Arabidopsis male meiocytes, we hypothesize that actin serves a comparable role in spindle positioning, particularly during meiosis II. In Arabidopsis male meiosis II, spindles are physically separated by the organelle band (Brownfield *et al*., 2015). We propose that the organelle band provides passive separation, while actin filaments contribute actively to spindle positioning. To investigate this, we analyzed the *jas* mutant, in which the organelle band is disrupted. In *jas* male meiocytes at metaphase II, actin filaments invade the middle zone, correlating with closely spaced or fused spindles. Notably, inhibiting actin polymerization chemically or expressing a dominant‐negative *ACTIN8* partially restored spindle separation, confirming that actin directly influences spindle dynamics by driving positioning.

## Results

### Reduced spindle distance in *jas* is associated with central actin invasion

**Fig. 1:**
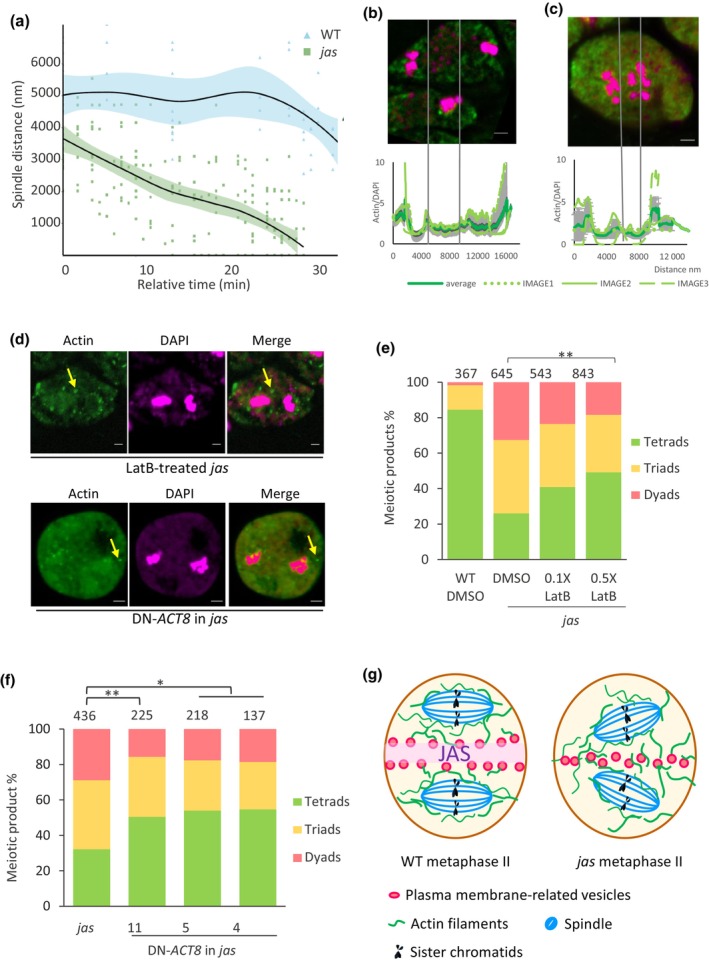
Actin filaments drive spindle positioning during male meiosis II in Arabidopsis. (a) Spindle distance dynamics in wild‐type (WT) and *jas*. In WT, spindle distances remain stable (4000–6000 nm) until anaphase II. In *jas*, spindle distance decreases continuously from *c*. 4000 nm to less than 1000 nm after metaphase II. Shading associated with the lines indicates the confidence intervals. *X*‐axis, relative time (min); *Y*‐axis, spindle distance (nm). Data represent mean ± SD from three biological replicates. (b) Actin localization in WT metaphase II cells. Actin filaments (fluorescent signal intensity line plot) are excluded from the organelle band and localized to peripheral cytoplasm. Gray shading indicates SD across three independent images. Bar, 2 μm; green color, actin; red color, DAPI. (c) Disrupted actin patterning in *jas* metaphase II cells. Actin filaments invade the middle zone (arrowheads). Line plot shows uniform actin distribution across the cytoplasm. Gray shading, SD across three images. Bars, 2 μm; green color, actin; red color, DAPI. (d) Actin filament disruption assays. Actin inhibitors (Latrunculin B, LatB) and DN*‐ACT8* expression fragment actin into punctate structures, shown in the yellow arrow, confirming functional inhibition of filament polymerization. Bars, 2 μm; green color, actin; red color, DAPI. (e) Latrunculin B (LatB) rescues *jas* meiotic defects dose‐dependently. LatB treatment (0.5×) significantly increases tetrad formation (25% in DMSO vs 50% in 0.5× LatB; **, *P* < 0.01, chi‐squared test) and reduces dyads/triads (75–< 50%). (f) DN‐*ACT8* expression partially restores normal meiosis in *jas*. Three independent DN‐*ACT8* transgenic lines exhibit an increased tetrad frequency (*c*. 50%) compared to *jas* (*c*. 30%) (**, *P* < 0.01; *, *P* < 0.05; chi‐squared test). (g) Model of spindle positioning. In WT, the organelle band and actin together maintain spindle separation. In *jas*, the disruption of the organelle band allows actin invasion, driving spindle convergence. Plasma membrane markers (dashed lines) remain localized to the middle zone in *jas* while the organelle band is disrupted. DAPI, 4′,6‐diamidino‐2‐phenylindole.

Spindle separation in male meiosis II is essential for ploidy reduction in male germline development. While it is known that the organelle band is important for spindle separation in meiosis II, we wondered whether the organelle band solely determines the spindle positioning. To test this hypothesis, we used the Arabidopsis wild‐type Col‐0 (WT) and the *jas* mutant with a disrupted organelle band. If the organelle band exclusively governs spindle positioning, spindles should be accurately aligned through organelle band‐mediated separation in WT but randomly positioned when the organelle band is disrupted in *jas* (Supporting Information Fig. S1A). We observed spindle position dynamics using a Tubulin‐GFP (TUB‐GFP) marker line under light‐sheet microscopy. In WT, the distance between two spindles remained relatively stable during meiosis II, *c*. 5000 nm (*n* = 13), with a slight decrease at very late stages, likely associated with the dynamic changes in tubulin (Fig. 1a, Videos S1, S2). This stability confirmed that spindle positioning is precisely regulated in WT. To further explore this, we examined spindle distribution in the *jas* mutant, which lacks an intact organelle band. Unlike WT, spindles in *jas* gradually moved closer to the middle zone during meiosis II (*n* = 16, Fig. 1a, Videos S3, S4). Hence, the spindle at metaphase II is not randomly positioned but intentionally driven to the middle zone in *jas*. This observation indicates that the organelle band is not the sole determinant of spindle positioning in male meiosis II and supports the presence of an additional mechanism driving spindles.

Although spindle positioning in cell division is not well understood in plants, actin is known to influence spindle positioning and movement in mammalian oocytes (Azoury *et al*., 2008; Li *et al*., 2008; Schuh & Ellenberg, 2008; Field & Lenart, 2011; Mori *et al*., 2011; Mogessie *et al*., 2018). We observed actin distribution in Arabidopsis male meiosis in WT and *jas* (Fig. S1B–M). In WT at metaphase II, actin distributed in the cytoplasm but was excluded from the middle zone, potentially due to the presence of the organelle band at metaphase II (Fig. 1b). The light intensity ratio of actin vs DAPI showed peaks around spindles, suggesting an assembly of actin in the cytoplasm, excluding nuclei and organelle band in WT (Fig. 1b). In contrast to WT, actin invaded the middle zone in *jas* (Fig. 1c), where the organelle band is disrupted. The peak of the light intensity ratio appeared at the middle zone (Fig. 1c). The redistribution of actin to the middle zone in *jas* mutants, coupled with spindle rejoining, indicates that actin functions in positioning the spindle during male meiosis II.

### Actin drives spindle position in male meiocytes

The localization of actin around spindles and its invasion into the middle zone in *jas*, where closed or fused spindles (the shortest distance between spindles is less than 2000 nm) are observed, suggests that actin plays a crucial role in spindle positioning, working alongside the organelle band. To investigate this role, we inhibited actin polymerization using Latrunculin B (LatB). After 24 h of treatment, actin filaments were disassembled (Fig. 1d). In *jas* meiocytes, abnormal meiotic products (dyads and triads) decreased from 70% in untreated samples to 60% with 0.1 μM LatB and further to 50% with 0.5 μM LatB (Fig. 1e). These results suggested that inhibiting actin polymerization significantly rescues the phenotype of closely assembled spindles in *jas* (chi‐squared test, **, *P* ≤ 0.01 represents dramatic significance, *, *P* ≤ 0.05 represents significance, ns *P* > 0.05 represents no significance). To further confirm that actin directly influences spindle positioning in male meiocytes, we expressed meiosis‐specific dominant‐negative *ACTIN 8* (DN*‐ACT8*) that can inhibit actin polymerization in the *jas* background. Actin dye staining of DN‐*ACT8* meiosis at metaphase II revealed disrupted actin filaments, which appeared as dot‐like structures (Fig. 1d). The actin staining result of metaphase II in DN‐*ACT8* is similar to that observed after LatB treatment. This confirmed the inhibition of actin polymerization during meiosis in DN‐*ACT8* plants. Like the effects of LatB treatment, DN‐*ACT8* transgenic lines in *jas* exhibited significantly fewer dyads and triads (50%) compared to *jas* (70%) (Fig. 1f). Together, our results demonstrate the role of actin in spindle positioning in *jas*.

## Discussion

Our results reveal that actin filaments actively contribute to spindle positioning during meiosis II in Arabidopsis, working alongside the organelle band to ensure proper spindle positioning. In WT, actin filaments surround the spindle at metaphase II and fill in the space between the organelle band and the plasma membrane, likely stabilizing the position of spindles (Fig. 1b). As the meiotic cell cycle progresses, actin dynamically associates with spindles from metaphase I until anaphase II (Fig. S1B–G). The absence of actin filaments at the organelle band was also observed in other plants, like Magnoliaceae (Dinis & Mesquita, 1993). In *jas*, where the organelle band is disrupted, actin filaments invade the middle zone, drawing spindles closer together and leading to abnormal meiotic products (Fig. S1H–M). Chemical inhibition of actin polymerization or expression of a dominant‐negative *ACTIN8* partially restores spindle separation, demonstrating that actin plays a direct role in spindle location.

In meiosis of higher plants, the absence of MTOCs not only provides more mutation possibilities but also brings meiotic errors. In female meiosis, the meiotic driving force comes from chromosomes and spindle rather than centrosomes, but the mechanism is not very well studied (Finseth, 2023). In mammalian oocytes, a similar mechanism was observed across species such as mice (Longo & Chen, 1985), human beings (Kim *et al*., 1998), pigs (Sun *et al*., 2001), and cows (Kim *et al*., 2000). In these cells, actin filaments surround the spindle and anchor it to the cell cortex, ensuring precise positioning and efficient relocation during meiosis. Similarly, our previous results reveal that the organelle band contains plasma membrane‐derived proteins (Brownfield *et al*., 2015), likely internalized via endocytosis, which may interact with actin to stabilize the spindle position, suggesting a parallel to the oocyte system. Further supporting this model, our previous work shows that, in *jas*, despite the absence of an intact organelle band, these plasma membrane‐derived proteins persist in the middle zone between the spindles (Piskorz *et al*., 2024). Without the physical barrier of the organelle band, the interaction between these proteins and actin filaments may generate a pulling force, causing the spindles to move centrally (Fig. 1g). Consistently, disrupting actin polymerization partially prevents this inward movement, reinforcing the role of actin in spindle positioning. Although the current knowledge of functional interaction between actin and spindle depends on advanced investigation techniques. These parallels between plant and animal meiosis underscore the evolutionary significance of actin‐mediated regulation, highlighting its conserved role in orchestrating successful cell division across species.

## Materials and Methods

### Plant material and growing conditions

*Arabidopsis thaliana* (L.) Heynh seeds of T‐DNA insertion mutants were obtained from the Nottingham Arabidopsis Stock Centre (NASC). The *jas* allele (SAIL_813_H03) and the WT *Arabidopsis* ecotype Col‐0 were used in this experiment, except as specially stated. *Arabidopsis* plants were grown under controlled conditions. Seeds were stratified for 3 d at 4°C and then germinated on plates containing ½‐strength Murashige & Skoog medium under a 16 h : 8 h, light : dark photoperiod at 21°C with a light intensity of 110 μmol s^−1^ m^−2^ and 70% relative humidity. After 10 d, seedlings were transferred to soil and grown in a growth chamber under identical photoperiod and temperature conditions.

### Microscopic analyses

To count the meiotic products, whole inflorescences were fixed in Carnoy's fixative (ethanol : acetic acid 3 : 1) for at least 3 h at room temperature. After two washes with 1× PBS, two to three of the largest white buds were dissected under a stereoscope, and the anthers were cut into pieces using sharp needles or a razor blade. The meiotic products were stained with 0.1–0.2% toluidine blue solution, viewed at 10× magnification under a stereoscope, and manually counted.

The F‐actin staining kit (ab112127; Abcam, Cambridge, UK) was used to stain F‐actin. To visualize actin, inflorescences undergoing meiosis were fixed in a 4% formaldehyde solution (v/v) for 20 min at room temperature. The samples were washed with the staining buffer from the kit and dissected as fast as possible. The anthers were stained by the F‐actin dye in a humid dark chamber for 20 min and then carefully washed away twice by the 1× dilution buffer in the F‐actin staining kit with a needle syringe. At the last wash, a small drop of buffer (*c*. 10 μl) was left to dissolve the 10× DAPI (4′,6‐diamidino‐2‐phenylindole) stock solution (0.2 μg ml^−1^ in H_2_O), with occasional dissecting using a razor blade or gentle squashing with a coverslip to flatten the sample. Imaging was performed using a Zeiss LSM780 confocal microscope equipped with a C‐Apochromat 40×/NA 1.2 water immersion objective. The intensity of light was measured by the ‘profile’ function in ZEN 3.4, and the relative intensity of the actin signal was calculated by the following formula:

$$\;\text{Actin}/\text{DAPI}\;=\log_{2}\left(\frac{A_{\lambda }=488\;\mathrm{on}\;\text{meiotic cell}-A_{\lambda }=488\;\mathrm{on}\;\text{background}}{A_{\lambda }=405\;\mathrm{on}\;\text{meiotic cell}-A_{\lambda }=405\;\mathrm{on}\;\text{background}}\right)$$

(A represents the profile number read from ZEN software. *A*_λ=488_ represents the profile number exited by the 488 nm layer, whereas *A*_λ=405_ represents the profile number exited by the 405 nm layer.) The background profile was measured outside of the cell structure on the selected images.

### Inflorescence culture and inhibitor treatment

The main stems of 6‐wk‐old Arabidopsis inflorescences were cultured on medium comprising 200 ml of 1× MS salts, 3% sucrose (w/v), and 1× vitamins. The pH was adjusted to 6.0 with KOH, and the medium was autoclaved at 121°C for 15 min before use. Inhibitors were prepared as a master mix. LatB (Sigma‐Aldrich) was dissolved in DMSO to create a 1 mM stock solution. The stock solution was stored at −20°C and fully dissolved before the experiment. Inflorescences were treated with 0.1 and 0.5 μM LatB. Control meiocytes were treated with DMSO. The prepared treatment medium was aliquoted into 0.5‐ml Eppendorf tubes and sealed with a sheet of adhesive foil to prevent contamination. A needle was used to create a hole in the middle of each tube. Inflorescences in excellent condition were selected, carefully cut with a blade, and immediately inserted into the hole in each 0.5‐ml Eppendorf tube for treatment. The culture system was incubated under standard conditions for 24 h. After this incubation period, the treated inflorescences were fixed and analyzed.

### Lightsheet fluorescence microscopy

Lightsheet Fluorescence Microscopy (LSFM) was performed using a Lightsheet 7 (Carl Zeiss GmbH) equipped with two pco.edge 4.2 sCMOS cameras (PCO AG) according to previous reports (Ovečka *et al*., 2015; Valuchova *et al*., 2020; Feng *et al*., 2023) with minor modifications. Approximately 0.5 mm Arabidopsis flower buds were carefully detached from the inflorescence, dissected to remove outer sepals, and inserted into 1.5 mm (inner diameter) glass capillaries (size 3, green mark; 701908; Carl Zeiss GmbH) containing 1% low melting agarose (A9045; Sigma‐Aldrich) prepared in distilled water. Once fitted in the microscope, the agarose cylinder containing the sample was extruded into the imaging chamber filled with distilled water. Temperature in the chamber was set to 21°C, and no artificial light source was supplemented. Imaging was done with 20× (W Plan‐Apochromat 20×/1.0) detection and 10× (LSFM 10×/0.2 foc) illumination objectives and 2.0× zoom. Excitation was done with 0.8% 488 nm laser line. Dual‐side illumination was chosen, and pivot scan was turned on to reduce shadowing. Z‐stacks covering the whole anther were taken every 45 s. Images were acquired with ZEN Black 3.1, and processing (selection of single z‐stack, drift correction where needed, ROI selection, movie export) was done with ZEN Blue 3.4 (both from Carl Zeiss GmbH).

### Plasmid construction

The DMC1 promoter was selected to drive the expression of *ACTIN8* (*ACT8*). The DMC1 promoter fragment and the *ACT8* coding sequence were amplified and cloned into the PGWB501 vector. Site‐directed mutagenesis was performed to change the E_272_ to K_272_ on *ACT8* using the Mut Express II Fast Mutagenesis Kit V2 (Vazyme, Nanjing, China). The accuracy of the construct was confirmed by Sanger sequencing before plant transformation.

## Competing interests

None declared.

## Author contributions

YM and HJ conceived and designed the experiments and wrote the manuscript. YM executed the experimental procedures. Both authors discussed the results and commented on the manuscript.

## Supporting information


**Fig. S1** The localization of spindle and actin filaments during meiosis in WT and *jas*.

**Video S1** The time‐lapse video of the TUB‐GFP signal in WT during meiosis in anther.

**Video S2** The time‐lapse video of the TUB‐GFP signal in WT during meiosis, zoomed into one cell.

**Video S3** The time‐lapse video of the TUB‐GFP signal in *jas* during meiosis in anther.

**Video S4** The time‐lapse video of the TUB‐GFP signal in *jas* during meiosis, zoomed into one cell.
Please note: Wiley is not responsible for the content or functionality of any Supporting Information supplied by the authors. Any queries (other than missing material) should be directed to the *New Phytologist* Central Office.

## Data Availability

The data that support the findings of this study are available in the article, in Fig. S1 and Videos S1–, S4.
