# Supplementary material for: Actin filaments drive spindle positioning in Arabidopsis meiosis II
Source: New Phytol. 2025 Sep 29;248(5):2187–91. doi: 10.1111/nph.70625 (PMC12589714; doi:10.1111/nph.70625)
Supplement: Supplementary file 1 — Fig. S1 The localization of spindle and actin filaments during meiosis in WT and jas. [file NPH-248-2187-s004.pdf]

New Phytologist Supporting Information
Article title: Actin filaments drive spindle positioning in Arabidopsis meiosis II
Authors: Yingrui Ma, Hua Jiang
Article acceptance date: 15 September 2025

Figure S1

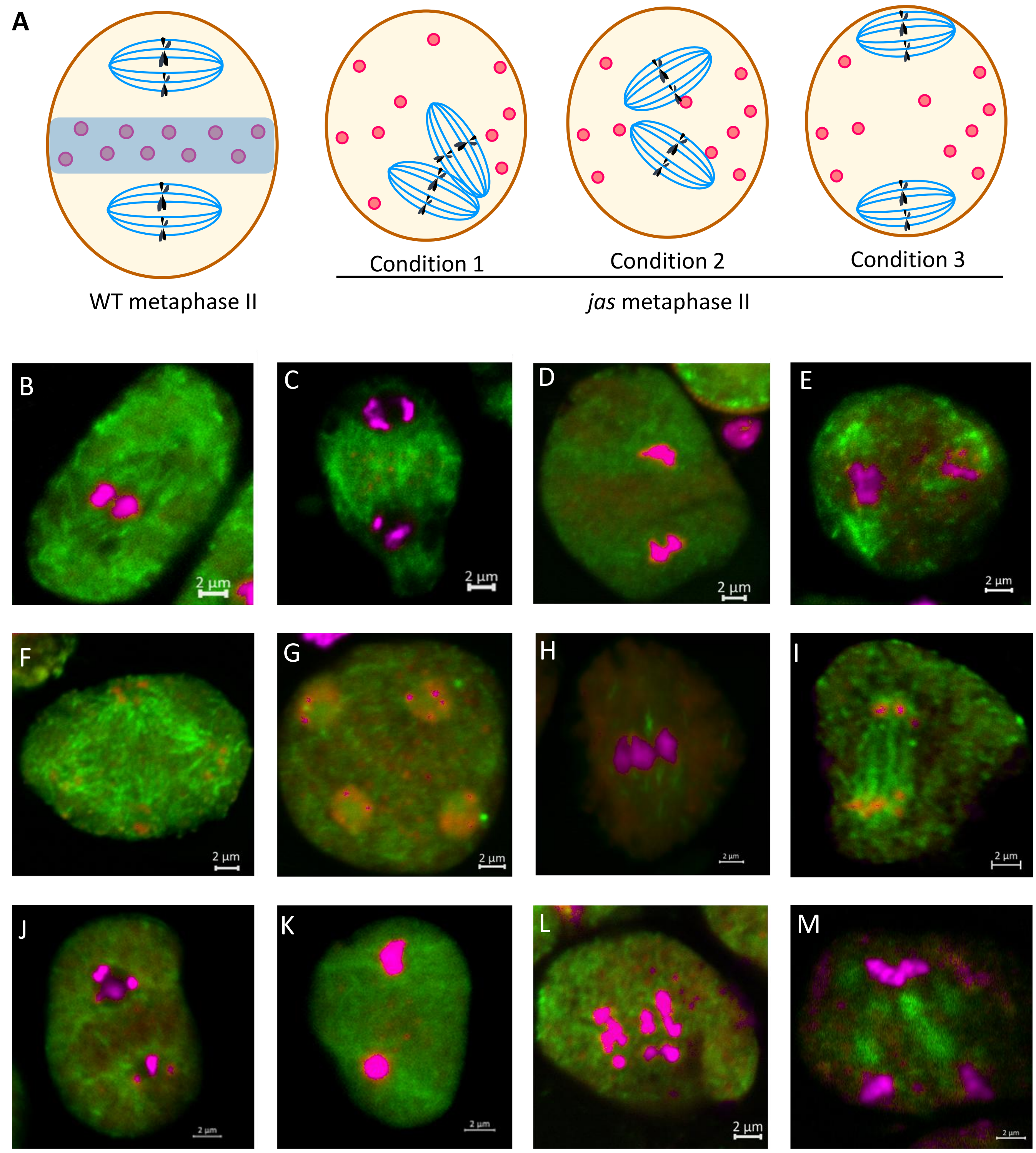

Figure S1 (A) Three possible spindle location conditions in the random localized spindle model in *jas*. (B to M) Dynamic actin filaments during meiosis in WT and *jas*. (B-G) WT meiosis, (H-M) *jas* meiosis. (B) metaphase I, actin is associated with the spindle and also the cytoplasm. (C) interphase. (D) early metaphase II, actin is located outside of the organelle band. (E) late Metaphase II, actin colocalize with spindles and is also located in the cytoplasm outside of the organelle band. (F) anaphase II. (G) telophase II. (H) metaphase I in *jas*. (I) anaphase I in *jas*, actin colocalizes with the spindle and is also located in the cytoplasm. (J) interphase. (K) early metaphase II, actin is located around the spindle and also invades into the organelle band. (L) metaphase II, actin is located around the spindle and also invades into the organelle band. (M) anaphase II, actin is associated with the spindle. Scale bar size: 2µm. Green: actin dye. Red: DAPI.
